# Computational analysis of translational readthrough proteins in Drosophila and yeast reveals parallels to alternative splicing

**DOI:** 10.1038/srep32142

**Published:** 2016-08-26

**Authors:** Rita Pancsa, Mauricio Macossay-Castillo, Simone Kosol, Peter Tompa

**Affiliations:** 1 Flanders Institute for Biotechnology (VIB), Structural Biology Research Center, Vrije Universiteit Brussel, 1050 Pleinlaan 2, Brussels, Belgium; 2 Institute of Enzymology, Research Centre for Natural Sciences of the Hungarian Academy of Sciences, 1117 Budapest, Hungary.

## Abstract

In translational readthrough (TR) the ribosome continues extending the nascent protein beyond the first in-frame termination codon. Due to the lack of dedicated analyses of eukaryotic TR cases, the associated functional-evolutionary advantages are still unclear. Here, based on a variety of computational methods, we describe the structural and functional properties of previously proposed *D. melanogaster* and *S. cerevisiae* TR proteins and extensions. We found that in *D. melanogaster* TR affects long proteins in mainly regulatory roles. Their TR-extensions are structurally disordered and rich in binding motifs, which, together with their cell-type- and developmental stage-dependent inclusion, suggest that similarly to alternatively spliced exons they rewire cellular interaction networks in a temporally and spatially controlled manner. In contrast, yeast TR proteins are rather short and fulfil mainly housekeeping functions, like translation. Yeast extensions usually lack disorder and linear motifs, which precludes elucidating their functional relevance with sufficient confidence. Therefore we propose that by being much more restricted and by lacking clear functional hallmarks in yeast as opposed to fruit fly, TR shows remarkable parallels with alternative splicing. Additionally, the lack of conservation of TR extensions among orthologous TR proteins suggests that TR-mediated functions may be generally specific to lower taxonomic levels.

Translational readthrough (TR), also referred to as stop codon readthrough, is a recoding mechanism that changes the traditional flow of biological information^1^. In case of TR, the translating ribosome decodes the stop codon as an amino acid by allowing one of the natural nonsense suppressor tRNAs to interact with it^2^ before protein release factors can terminate translation. Thus, with a certain frequency, the ribosome continues to translate the mRNA until it reaches the next in-frame stop codon, thereby giving rise to C-terminally extended proteins.

For a long time, TR was only attributed to viruses^3^, where the mechanism is frequently employed to optimize coding capacity, to produce small amounts of essential proteins^4^ or to ensure an ideal ratio between certain proteins (e.g. Gag and Pol in many retroviruses^5^). Later, TR has also been described in bacteria^6^,^7^ and eukaryotes^8^,^9^,^10^,^11^,^12^,^13^,^14^,^15^,^16^,^17^,^18^,^19^. Some TR-extended protein forms were shown to function differently from their non-extended counterparts, like PDE2^14^ and IMP3^9^,^13^ in yeast, the hdc (headcase) gene of *D. melanogaster*^16^,^17^ or myelin protein zero (MPZ)^19^ and vascular endothelial growth factor A (VEGFA)^20^ in human. Functional difference may entail differential localization, into the peroxisomes^10^,^21^,^22^, or their production in a tissue-specific manner^15^.

The immediate sequence context of the stop codon has an important role in promoting TR^22^,^23^,^24^. Based on this context and other features, there have been attempts to predict TR in yeast^13^,^14^, fruit fly^25^ and human^21^,^26^.

The comparative genomics analysis of 12 Drosophila genomes^27^ suggested abundant stop codon readthrough based on evolutionary signatures. In a subsequent computational analysis, using PhyloCSF^28^ which is a comparative genomics approach to detect protein-coding type evolutionary constraints within nucleotide sequence alignments, Jungreis *et al*. confirmed the in-frame continuation of protein-coding signatures after the annotated stops right until the subsequent in-frame stop codons in hundreds of *D. melanogaster* genes. The identified cases were manually filtered to yield 283 genes, for which the observed conservation patterns could be best explained by TR^29^; these will be hereinafter referred to as evolutionarily conserved TR cases. A few of these TR products were also validated experimentally. Genomic analysis of the six closest relative species of *D. melanogaster* suggested that about 100–200 genes evolved into readthrough genes after the divergence of the 12 species, without specific details of these affected candidate genes. Jungreis *et al*. also identified a few putative TR genes in other insects, *Caenorhabditis elegans,* and human. In Saccharomyces and Candida species, on the other hand, they could not identify genes with unambiguous signatures of readthrough, even though a few cases have been described previously^13^,^14^. This has led to the conclusion that in metazoans TR is mainly abundant in Arthropoda, and that the evolutionary conservation of TR extensions and the positions of the surrounding stop codons indicate their functional importance and evolutionary advantage.

Later, more than three hundred new TR genes were identified by ribosome profiling in *D. melanogaster* early-stage embryonic cells and S2 cells^30^. Surprisingly, Dunn *et al*. could only confirm ~15% of the previously identified^29^ 283 evolutionarily conserved TR cases, leading to the suggestion that many genes could undergo TR in other developmental stages not represented in the experiments, or a larger sequencing depth is required to detect them. In most of the newly identified TR extensions, no strongly conserved protein coding signatures were detected by PhyloCSF^28^ using the alignments of the 12 Drosophila species. Unfortunately, the extensions were not analysed on a smaller set of more closely related species. Lack of conservation of the novel extensions may mean that they are not subject to TR in some Drosophila species, or that they have undergone extensive sequential diversification, for instance as a result of frameshift-inducing insertions/deletions. This raises the question if these novel cases are selectively neutral, non-functional, or if they became positively selected only later in the melanogaster lineage. Dunn *et al*. tried to distinguish between these possibilities and found that 1) the novel extensions have an intermediate nucleotide character between coding regions and distal 3′ UTRs, 2) novel extensions show a significant preference for synonymous single nucleotide polymorphisms (SNPs), above the background level of distal 3′ UTRs, but below that of conserved extensions, and 3) ~62% of the novel extensions showed biologically significant readthrough rates based on those of the conserved extensions. They concluded that at least a subset of the novel extensions have come under evolutionary selection within the melanogaster lineage (which could well match those 100–200 genes that were identified when only 7 Drosophila species were compared), while others may have undergone diversifying selection or have been selectively neutral. Additionally, for a few candidate proteins, Dunn *et al*. observed remarkably different readthrough frequencies in the investigated cell types, providing further evidence for the regulated nature of TR. By analysing previously published ribosome profiling data, they also identified a few dozen human and yeast TR genes, which showed biologically relevant readthrough rates.

In yeast, several mechanisms are implicated in the controlled extension of proteins by TR^31^. Both genetic and epigenetic regulatory mechanisms may lead to abundant readthrough in [PSI^+^] strains, where the translation termination factor Sup35p/eRF3 adopts a prion form, conferring a beneficial phenotype under stress conditions^32^. Based on recent ribosome profiling experiments performed by Artieri and colleagues, many yeast genes are likely to undergo TR even in [PSI^−^] strains^33^. They studied two species (*S. cerevisiae* and *S. paradoxus*), and proposed both conserved and species-specific TR genes^33^.

Although these comprehensive analyses identified many TR candidate genes, they failed to provide sufficient understanding of the functional-evolutionary forces that maintain TR in eukaryotes, also hampered by the lack of structures available for proteins with TR extensions from any species. Similarly to alternative splicing, TR most probably provides an economic way of increasing proteome versatility by the regulated incorporation of additional functional modules at the C-termini of proteins^29^,^30^,^34^. Alternative splicing has a central role in the tissue- and developmental stage-specific proteome diversification of higher eukaryotes. This is mainly achieved through alternatively spliced (for instance, tissue-specific) exons that often encode intrinsically disordered protein regions^35^,^36^. Disordered protein regions function as an ensemble of different conformations^37^ and usually fulfil regulatory roles^38^. Alternatively spliced disordered regions are usually very rich in short linear interaction motifs^35^,^36^ and hence show high potential for rewiring cellular interaction networks^39^. TR is also similar and at the same time complementary to leaky scanning, a mechanism for N-terminal protein diversification by alternative translation initiation sites that can give rise to protein forms with altered localization or biological functions^40^.

Our premise is that an in-depth structure-function analysis of TR proteins could promote our understanding of the functional benefits, if any, and evolutionary secrets of TR^41^ as well as its relation to alternative splicing. For instance, the lack of annotated protein domains in the C-termini of TR proteins could explain their increased tolerance against the potentially destabilizing effects of TR extensions. A comparison of the biological processes favoured by TR proteins in different species could point to species-specific functional specializations. Additionally, a better view on the structure and interaction potential of TR extensions could help elucidate their functional roles. To fill this gap, we report here the results of a comprehensive computational study on *D. melanogaster* and *S. cerevisiae* TR proteins and extensions.

## Materials and Methods

### Collection of protein sequences and extension regions of TR candidate genes

The evolutionarily conserved *D. melanogaster* TR candidate genes^29^ and the *D. melanogaster* and *S. cerevisiae* candidates observed by ribosome profiling^30^ were compiled from datasets published in the latter study. For the 558 *D. melanogaster* candidates, the transcript identifiers were checked in Ensembl 74^42^. The sequences of 28 candidates whose identifiers were not found in the database were searched in the Ensembl 74 *D. melanogaster* proteome. Seven hits that showed a perfect match with an entry of equal length in the database were kept under the new identifiers, but the others were excluded. Finally, 537 readthrough cases were subjected to further analyses.

The 30 *S. cerevisiae* TR proteins reported by Dunn *et al*. were also retrieved and complemented by TR proteins suggested by Artieri *et al*.^33^. From the latter study only those *S. cerevisiae*-specific and conserved TR candidates (149 entries) were adopted that fulfilled the strict criteria of showing ≥5 reads in the extension region in both combined hybrid and combined parental replicates of *S. cerevisiae*. 7 cases in which the first three in-frame extension codons contain an AUG (start) codon that could facilitate reinitiation were excluded. After merging the two datasets, we obtained 165 unique *S. cerevisiae* TR proteins that were subjected to further analysis.

For human, the 46 readthrough candidate genes suggested by the two studies were not sufficient for a reliable statistical analysis of structural properties, and thus human TR candidates were not used in this study.

The residues corresponding to stop codons are ambiguous (depend on the inserted nonsense suppressor tRNA^2^), they are therefore represented by unknown residues (X). Regions derived from double readthrough were not included in our analysis.

### Nomenclature of the datasets used for large-scale statistical analyses

The proteomes of *D. melanogaster* and *S. Cerevisiae* were retrieved from Ensembl 74. The resulting reference proteomes contain all protein isoforms (26916 and 6692, respectively) that could be used for predictions by all the applied methods (proteins longer than 10000 residues were excluded due to the limitations of the PSIPRED method). The term “TR candidate” refers to the normal protein forms, while the term “extended TR candidate” refers to the TR-extended forms. The following datasets were created for both species: 1) NonTRC contains the proteome excluding all products of TR candidate genes, 2) TRC contains the TR candidate proteins (537 and 165, respectively) without extensions, 3) TRC_C includes the C-terminal regions of TR candidate proteins of equivalent length to their extensions 4) TRC_C30 contains the C-terminal 30 residue segments of the TR candidate proteins, and 5) TRC_E contains the extensions. Due to a few cases, where the extensions were longer than their candidate proteins, a slight difference between the total segment lengths of the equivalent TRC_E and TRC_C datasets might occur. The TRC_C dataset was used as a reference for statistical comparisons just like 6) the RAND_C dataset, that was assembled by selecting a non-candidate protein of similar (±5%) length for each extended TR candidate from the proteome, and taking its C-terminus of equivalent length to the corresponding TR extension. This procedure ensured that segments occupied C-terminal positions, and represented similar fractions of their proteins as the extensions. After filtering these datasets for a minimum extension length of 25 residues, they were distinguished by the suffix “_L” attached to their names. The *D. melanogaster* candidates detected by ribosome profiling were also filtered for a minimum readthrough rate of 1.2% of the translation rate of the corresponding CDS, a threshold of biological relevance suggested by Dunn *et al*. based on the readthrough rates of evolutionarily conserved TR genes. These were then merged together with the evolutionarily conserved cases to obtain the group of biologically relevant TR proteins that was distinguished by the suffix “_BR” in its name.

### Prediction of structural properties and binding motifs

For proteins/segments we determined the following structural measures: 1) the fraction of disordered residues, which score ≥0.5 by IUPred^43^, 2) the fraction of residues in low sequence complexity regions predicted by SEG^44^, 3) the fraction of residues in any secondary structure type (helix or extended) assigned by PSIPRED v3.35 (without building PSI-BLAST profiles)^45^, and 4) the fraction of residues in PfamScan-identified A-type Pfam entities (Pfam release 27)^46^. All four Pfam entity types were accepted (domains, families, repeats and motifs, hereafter collectively referred to as Pfam entities). The following measures of function/interaction capacity were also applied: 1) the fraction of residues in disordered binding sites^47^, 2) the number of potential eukaryotic linear motifs (ELMs) that overlap with disordered regions (a filter also applied by the ELM browser^48^, however, here a reduced threshold of disorder probability (≥0.4) was used based on Fuxreiter *et al*.^49^), and 3) the number of ELMs that also overlap with disordered binding sites. The Anchor method^47^ was used for predicting disordered binding sites, i.e. regions prone to fold up on binding to a protein partner, as well as for detecting ELM patterns in the extended TR candidates (species-specific ELM sets were applied). ELMs were considered as part of an extension region if they had at least one residue overlap (incomplete ELMs could be completed by even one extension residue).

We were led by several considerations when deciding on the applied methods: 1) IUPred is a widely used, freely available, locally applicable, fast disorder prediction method, which is often used for analysing whole-proteome data and also as a filter in ELM search^48^. Its prediction results are easy to understand and interpret due to the clear physical principles it relies on^43^. Also, since IUPred does not take sequence complexity into account when estimating disorder of a protein region, it can be used along with SEG without introducing redundancy into our measures. 2) SEG is based on a simple formula describing the compositional complexity of sequences using sliding windows^44^, it is widely applied for detecting low complexity regions in sequences. 3) PSIPRED is a frequently used, accurate, fast, and freely available secondary structure predictor. Also, it can be applied with sequence information alone (not using PSI-BLAST profiles). Although this option reduces accuracy somewhat, it enables predictions on proteome-scale data in reasonably short times, and allows the analysis of the structural properties of TR-extensions (which lack any homologues in databases) under conditions identical to their reference segments. 4) ANCHOR predicts disordered binding regions by relying on physical principles, hence it can complement sequence pattern-based interaction motif searches (like ELM search) and reduce their high false positive rates^50^, while potentially detecting interaction sites not (yet) described by motifs.

Most of the applied methods work with sequence windows, which allow the evaluation of the structural properties of residues in their natural sequence environment. To this end, we excised the values corresponding to the segments of interest from the prediction results of full-length proteins.

### Identification of orthologous protein pairs between the TR candidates of the two species

The full list of orthologous protein pairs between the two species was obtained from the Inparanoid v7 database^51^; we identified 12 orthologous pairs among our candidate proteins. The pairs were aligned by ClustalW, but in the majority of the resulting alignments the extensions (the X residues representing the stop codons) were not fitted. In these cases the extensions were separately aligned.

### Statistical evaluation of results

We used the GOrilla server^52^ to perform GO enrichment analysis of TR candidate sets. Since Jungreis *et al*. showed that TR mainly affects long transcripts in *D. melanogaster*, we performed the GO enrichment analysis of each TR protein set by using a reference set corrected for length. In this, a subset of the corresponding proteome was assembled by randomly selecting a non-candidate protein of length similar (±5%) to each TR candidate.

Our datasets are not normally distributed (by D’Agostino & Pearson omnibus normality test), so Mann-Whitney U test was applied for pairwise comparisons. We used Kruskal-Wallis test if more than two datasets had to be compared. If significant differences were detected, Dunn’s multiple comparison post-hoc test was performed to identify which datasets differ. Yates’ chi-square tests were applied for comparisons on the residue basis. If multiple structural properties determined in the same dataset were compared, Bonferroni correction was applied on the significance thresholds. Data handling was controlled by custom Perl scripts. GraphPad Prism 6 was used for statistical tests and figure preparation.

## Results

All the *D. melanogaster* and *S. cerevisiae* proteins proposed to undergo stop codon readthrough by comparative genomics analyses^29^, or ribosome profiling studies^30^,^33^ have been collected and subjected to a comprehensive structure-function analysis. We used several metrics to describe the properties of the proteins/segments: the fractions of 1) disordered (by IUPred), 2) low complexity (by SEG), 3) secondary structure (by PSIPRED), 4) Pfam entity (by PfamScan) and 5) disordered binding site (by Anchor) residues, and also, 6) the number of potentially functional ELMs (those that lie in predicted disordered regions). ELM patterns are degenerate and thus the computational detection of functional ELMs is affected by very high false positive rates. However, our reference datasets are defined so that they are similarly affected by this as the investigated extensions. We also take into account that functional ELMs tend to overlap with disordered regions^48^,^49^ and Anchor binding sites^50^. Nevertheless, without experimental evidence one cannot conclude on the functionality of individual motifs, so our motif-detection results only reflect tendencies in the interaction capacities of the investigated sequence regions. All information, including the predicted properties, of TR candidates and extensions are provided in Supplementary Tables S1 and S2 for *D. melanogaster* and *S. cerevisiae*, respectively. Extension ELMs were detected using Anchor and tested for overlapping disordered regions^48^,^49^ and binding sites^50^ (data presented in Supplementary Tables S3 and S4 for *D. melanogaster* and *S. cerevisiae*, respectively).

For both species, the structural properties of TR candidate proteins were compared to those of the non-candidates to see if they show any special character indicative of TR. Additionally, their last 30 residues were evaluated separately since TR-derived extensions affect the C-terminal regions. GO enrichment analyses using the GOrilla server were performed to identify the biological processes favoured by TR proteins (enriched GO terms are presented in Supplementary Tables S5 and S6 for *D. melanogaster* and *S. cerevisiae*, respectively). Furthermore, the TR-derived extensions were investigated from both structural and functional aspects and compared with two reference sets: 1) randomly selected protein C-termini of equal length, and 2) C-termini of equal length of the non-extended candidates. By this, their properties could be adequately evaluated with respect to their global and local (sequence) environments. The comparisons were performed using both the segments and their residues as units.

### Proteins undergoing readthrough in *D. melanogaster* are long, have structurally disordered C-termini and fulfil mainly regulatory roles

In this study, 537 *D. melanogaster* TR candidate proteins (TRCs) were investigated^29^,^30^. TRCs are longer (median of 510 residues vs. 456 residues; Mann-Whitney U test, p = 6.4E-03) than the rest of the proteome (NonTRC), and their C-terminal segments are significantly enriched in disordered and low complexity regions (Supplementary Figure S1).

Compared to their length-matched controls, TR proteins show a slight preference for nuclear localization (1.3 fold; p = 4.89E-7) and GO biological processes related to regulation (1.17 fold; p = 1.35E-7), including the regulation of gene expression (1.29 fold; p = 1.83E-5), metabolic processes (1.2 fold; p = 5.52E-5) and biosynthetic processes (1.28 fold; p = 4.69E-5). Also, they are enriched in developmental proteins functioning in anatomical structure morphogenesis (1.24 fold; p = 2.42E-4; see Supplementary Table S5).

### The structure-function properties of *D. melanogaster* TR extensions

**Figure 1:**
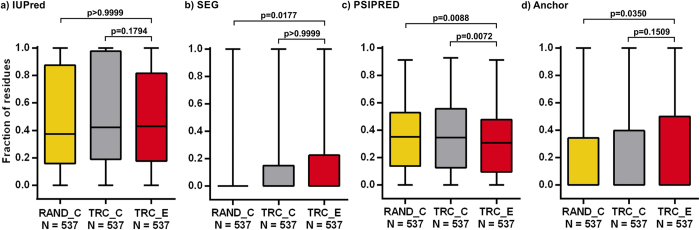
The structural and interaction properties of *D. melanogaster* TR extensions. The fractions of residues in predicted (**a**) disordered (by IUPred), (**b**) low complexity (by SEG), (**c**) secondary structure (by PSIPRED) and (**d**) disordered binding regions (by ANCHOR) were calculated for the TR extensions (TRC_E; red) and compared to those for the randomly selected C-termini (RAND_C; yellow) and the C-termini of the TR candidates (TRC_C; grey) using Kruskal-Wallis test coupled with Dunn’s multiple comparison post-hoc test. The p-values indicated above the box plots are adjusted according to the number of comparisons performed in each panel. The significance threshold is further decreased to p = 0.0125 by Bonferroni correction due to the multiplicity of properties compared.

The TR-derived extensions of *D. melanogaster* proteins vary in length from 3 to 555 residues with a median of 19 residues. They have absolutely no Pfam entities, implying that they are not the result of premature termination codons (PTCs), thus this property was not used for comparisons. They show reduced secondary structure content compared to the reference segment sets, but seemingly do not differ in the other properties investigated (Fig. 1).

**Figure 2:**
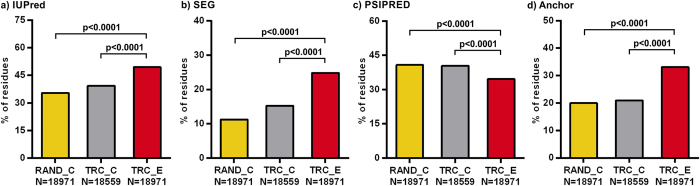
The structural and interaction properties of *D. melanogaster* TR extensions on a residue basis. The predicted properties of TR extensions (TRC_E, in red) were compared to those of the two reference segment sets, RAND_C in yellow and TRC_C in grey, using their residues as units. The numbers of (**a**) disordered, (**b**) low complexity, (**c**) secondary structure element and (**d**) disordered binding site residues were used for comparisons by Yates’ chi-square tests. In the statistical testing, the number of positively assigned extension residues (TR extensions contain 18971 residues in total) was used as observed value, while the product of the fraction of positively assigned reference residues and the number of extension residues was used as expected value for each property. The bars show the percent of positively assigned residues. The significance threshold is adjusted to p = 0.00625 using Bonferroni correction.

The residue-based analysis, however, uncovered that *D. melanogaster* extensions are depleted in secondary structure residues, and are highly enriched in residues of disordered, low-complexity and binding regions compared to both reference datasets (Fig. 2, Supplementary Table S7). This result is also supported by their amino acid composition, since extensions were found to be rich in certain disorder-promoting amino acids (Pro, Gln, Arg, His and Ser) compared to the SwissProt database^53^ and to a lesser extent also in comparison with the corresponding reference proteome (Supplementary Figure S2).

The contradictory results obtained from the segment- and residue-based comparisons indicate that structural bias cannot be accurately addressed for short extensions, since their predicted structural properties are defined by the large sequence windows applied by the corresponding methods. Since SEG estimates the complexity of 12-residue windows, it does not necessarily assign an extension of five residues with even four identical amino acids as low complexity. Also, the X residues representing stop codons definitely increase the complexity of all their corresponding SEG windows, although the actually inserted residues could also affect them oppositely. Due to these reasons, the comparisons were repeated for extensions of at least 25 residues in length. This length cut-off guaranteed that the predicted region corresponds to at least half of an IUPred (short) window.

**Figure 3:**
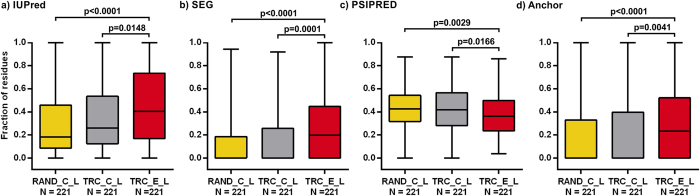
The structural and interaction properties of long *D. melanogaster* TR extensions. The fractions of residues in predicted (**a**) disordered, (**b**) low complexity, (**c**) secondary structure and (**d**) disordered binding regions of long (>25 residues) TR extensions (TRC_E_L, in red) were compared to those of the similarly filtered two reference segment sets, RAND_C_L (in yellow) and TRC_C_L (in grey) in the same way as explained in Fig. 1.

The comparison of long *D. melanogaster* protein extensions (221 extensions) to the randomly selected reference segments showed that these are highly disordered and show high interaction potential, while being depleted in regions with well-defined structural organization. Compared to the C-terminal segments of the TR candidates, the extensions were found to be significantly enriched in low complexity and disordered binding regions (Fig. 3). To make sure that the observed tendencies were not due to functionally irrelevant readthrough regions, we repeated the comparison using only those extensions that were identified based on evolutionary signatures or showed biologically relevant readthrough rates (≥1.2% of the translation rate of the corresponding CDS) in the ribosome profiling experiments performed by Dunn *et al*. The resulting set of 178 biologically relevant readthrough cases showed similar structural properties to the long extensions (Supplementary Figure S3).

**Figure 4:**
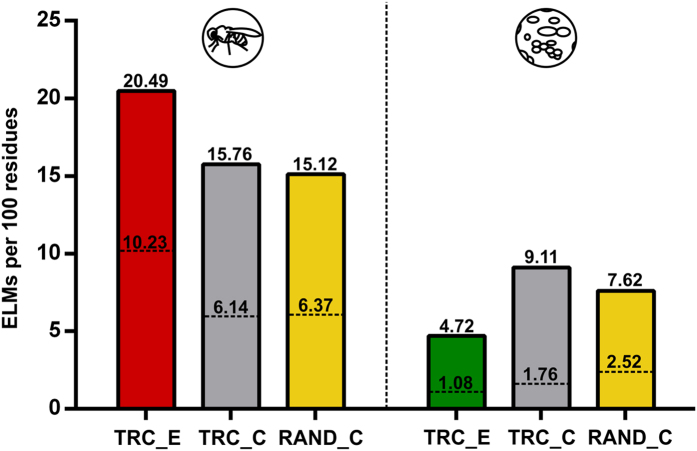
The interaction capacity of TR extensions. The numbers of potentially functional ELMs per 100 residues are shown for the extensions and the equivalent reference sets (RAND_C (in yellow) and TRC_C (in grey)) for both species. The height of the bars and the numbers on top indicate the number of potential ELMs (with at least one disordered residue ≥0.4 by IUPred). The dashed lines within the bars and the numbers on top indicate the number of potential ELMs also overlapping Anchor-predicted disordered binding sites.

*D. melanogaster* TR extensions contain more detectable linear motif patterns (ELMs) than the equivalent reference segments, further supporting an increased interaction capacity within extensions. In 537 extensions, 5497 ELM patterns were detected, more than two thirds of which overlap with disordered regions (20.5 potential ELMs per 100 residues), and 1941 also overlap with Anchor-predicted disordered binding sites^50^ (Fig. 4, Supplementary Table S8).

### *S. cerevisiae* TR proteins are short and are mainly involved in housekeeping processes

Merging and filtering of published ribosome profiling data resulted in 165 unique yeast TR candidate proteins^30^,^33^. In contrast to the *D. melanogaster* TR proteins, yeast TRCs are short in comparison to the rest of the yeast proteome (median of 258 residues vs. 362 residues; Mann-Whitney U test; p < 1E-04). In addition, they are enriched in Pfam entities (p < 1E-04), but do not differ in any of the other structural properties investigated. In contrast to *D. melanogaster*, the C-termini of *S. cerevisiae* TRCs are highly enriched in Pfam annotations (p < 1E-04), but they do not deviate from the reference datasets in other properties.

GOrilla identified yeast candidates to be preferentially localized in the ribosome (1.57 fold; p = 6.86E-4) and accordingly, to be often involved in translation (5.48 fold; p = 1.21E-10). Thus, in contrast to fruit fly TRCs, yeast TR proteins do not favour regulatory roles, but biosynthetic processes (Supplementary Table S6).

### The structural and functional properties of *S. cerevisiae* TR extensions

**Figure 5:**
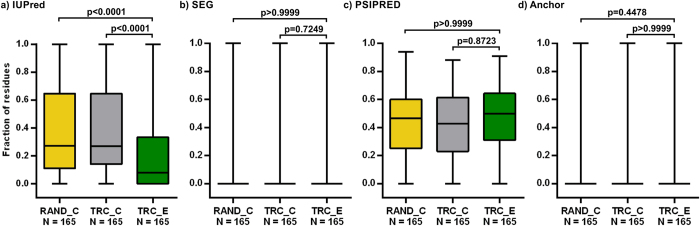
The structural and interaction properties of yeast TR extensions. The fractions of residues in predicted (**a**) disordered, (**b**) low complexity, (**c**) secondary structure and (**d**) disordered binding regions of yeast TR extensions (TRC_E, in green) were compared to those of two equivalent reference segment sets, RAND_C (in yellow) and TRC_C (in grey) in the same way as described in Fig. 1.

**Figure 6:**
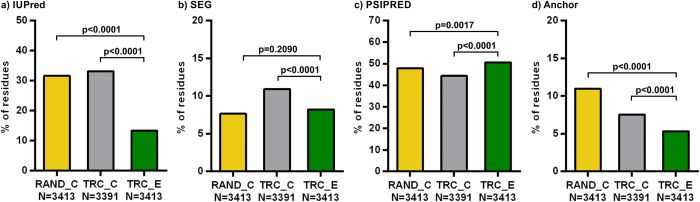
The structural and interaction properties of yeast TR extensions on a residue basis. The predicted properties of yeast TR extensions (TRC_E, in green) were compared to those of two reference segment sets, RAND_C in yellow and TRC_C in grey, using their residues as units (3413 extension residues in total). The numbers of (**a**) disordered, (**b**) low complexity, (**c**) secondary structure element and (**d**) disordered binding site residues were used for comparisons by Yates’ chi-square tests in the same way as in Fig. 2.

Yeast TR-derived extensions vary from 4 to 79 residues in length (median of 20), and are significantly less disordered than the equivalent reference C-termini (Fig. 5). Again, no Pfam entities were detected within the extensions. The extensions are highly depleted in residues residing in disordered regions and binding sites, while they are enriched in those located in secondary structure elements compared to both reference sets (Fig. 6, Supplementary Table S7). In agreement with their predicted structural properties, they are enriched in certain hydrophobic residues (Tyr, Leu, Ile, Phe and Cys) and depleted in some disorder-promoting ones (Ala, Gly, Asp, Glu and Gln compared to the SwissProt database and also to the corresponding reference proteome; Supplementary Figure S2). The same tendency is observed when only segments of at least 25 residues are analysed; even longer extensions lack disordered regions and binding sites, a tendency confirmed by the analysis of ELMs (Supplementary Figure S4, Fig. 4). Yeast TR extensions could not be filtered for relevant readthrough rates, since the vast majority of the data was adopted from Artieri *et al*. who provided the number of observed footprints for the extensions, but no readthrough rates.

In the 165 yeast TR extensions (3413 residues in total), 853 ELM patterns can be detected. Of these, only 161 overlap with disordered regions (4.7 potential ELMs per 100 residues) and 37 overlap with Anchor-type binding sites. Yeast extensions show much less capacity for interactions than *D. melanogaster* extensions (4.7 vs. 20.5 potential ELMs per 100 residues), even if the smaller number of species-specific ELMs used for detection (116 yeast ELMs vs. 180 fruit fly ELMs) is considered. More importantly, yeast extensions also contain less potential ELMs than the equivalent reference sets (Fig. 4, Supplementary Table S8).

### Structure-function properties of experimentally validated eukaryotic TR extensions

All the eukaryotic TR proteins have been collected that were experimentally verified, independently from large-scale studies. Their predicted properties (Table 1) were in agreement with our previous results; while proteins of fungi species (the first eight rows of Table 1) show very low disorder, binding site, and motif content in their extensions, *D. melanogaster* protein extensions are long and highly disordered with many embedded potential binding sites and ELMs. In the seventh column of Table 1, we listed those extension ELMs that are of relatively low probability (<1E-03) and hence are not very likely to occur by chance (for *D. melanogaster* only the numbers of such motifs are indicated, the detailed list is provided in Supplementary Table S9). Besides detecting most of the previously reported peroxisome targeting signals^10^,^21^,^22^, we have also detected other potential functional motifs in the extension regions, including nuclear localization signals and several PDZ domain recognition sites, among others. We suggest that the functionality of these motifs should be tested experimentally.

### Analysis of orthologous pairs of TR proteins

We identified 12 orthologous pairs among our TR candidates based on the Inparanoid v7 database^51^. To investigate whether TR of these genes is conserved between these species or independently appeared during evolution, we first aligned the orthologs and then investigated the conservation of sequence and potential functional motifs within their TR extensions. Although the overall sequence conservation was high within the CDSs, the starts of the extension regions were often not well-matched and thus were realigned separately. In each pair, TR extensions were remarkably less conserved than the corresponding CDSs. Most extensions were not detectably conserved at all and their lengths often showed large differences (Supplementary Figure S5). A few potentially conserved motifs (ELMs) were identified within five of the aligned extension regions; however most of them did not fulfil the criteria of overlapping structurally disordered regions or predicted disordered binding sites. Furthermore, most of them have very high probability scores according to the ELM database^48^, which means that they are degenerate, i.e. very likely to occur in any protein sequence by chance like phosphorylation sites, for instance. We could detect several of these even within our relatively short TR extensions. Disregarding motif types with high probability scores (>1E-03) resulted in only one orthologous pair with a conserved motif, while further decreasing this limit to 1E-04 would have completely eliminated the potential hits. In summary, neither sequence conservation nor conserved motifs provided evidence for the evolutionary conservation of the investigated TR extensions.

We also tried to evaluate the possible conservation of TR between the orthologs based on their conservation among closely related species. If TR was conserved between so distantly related species as yeast and fruit fly for these proteins, it would be reasonable to expect that it is also conserved among the different Drosophila species. Surprisingly, we found only one among the 12 fruit fly proteins (MBF1) that has been detected by comparative genomics by Jungreis *et al*. (Supplementary Figure S5). Although most yeast TR proteins were subject to TR in both yeast species investigated by Artieri *et al*.^33^, they had different, often frameshifted extension sequences.

## Discussion

Translational readthrough is one of the few known recoding mechanisms, in which the genetic information gets overridden during translation. It was initially described in viral genomes, where the associated advantages are clear. In eukaryotes, TR is much less understood, however the last few years brought several breakthroughs. TR turned out to be relatively abundant in certain eukaryotes^29^,^30^,^33^, such as insects and yeasts, and to be often subject to regulatory control^15^,^30^. Readthrough of stop codons can be conserved across closely related species^29^,^33^ but can also be species-specific^30^,^33^. Although the recognized number of such genes has increased remarkably, many key aspects of TR, such as the functional advantages provided by the resulting protein extensions, the evolutionary history of TR within eukaryotes, or its potential role in species differentiation, are still largely unknown.

Our premise was that TR-derived protein extensions could be very similar to the segments encoded by alternatively spliced or tissue-specific exons in terms of structural organization and functional roles. To test this hypothesis, we performed a comprehensive computational structure-function analysis of *D. melanogaster* and *S. cerevisiae* TR candidate proteins and extensions. Besides aiming at a better understanding of their potential functional roles, we also attempted to discover possible species-specific specializations of eukaryotic TR.

In Drosophila species, which have the highest number of reported TR genes among eukaryotes, TR extensions most probably fulfil similar functions to tissue-specific exons. Here, TR affects long, modular proteins with structurally disordered C-termini, which are often of low sequence complexity. The lack of well-structured regions at the C-termini of TR proteins could explain their tolerance to potentially destabilizing C-terminal extensions. Also, these characteristics probably increase the accessibility of extensions and thereby enable them to engage in interactions independently. On top of this, *D. melanogaster* TR proteins are mainly involved in regulatory roles, which justifies their need for the addition of interaction-prone segments specifically fine-tuning their functions in a temporally and/or spatially regulated manner. The structure-function properties and evolutionary conservation of TR extensions further support our hypothesis. Although sometimes reaching several hundred residues in length, the extensions contain no regions of homology to any PFAM entities. As judged by disorder prediction methods and their biased amino acid composition, a large fraction of fruit fly TR extensions, especially the longer ones, tends to be disordered and of low sequence complexity. At the same time, they are rich in disordered binding sites and short linear interaction motifs which, together with their structural properties, makes them ideal for effectively rewiring interaction networks. Many of the investigated extension sequences are conserved among Drosophila species^29^, which supports the hypothesis of their functioning through sequence motifs.

Yeast, on the other hand, is a unicellular organism, which utilizes only a rudimentary splicing-like mechanism in a few dozens of its genes^54^. Considering this, it is not surprising that in yeast TR is also much more restricted and lacks clear functional hallmarks. We found that TR in yeast mainly affects shorter than average proteins involved in basic housekeeping functions, like translation. This result shows that the findings of Jungreis *et al*. in *D. melanogaster* that TR affects mainly long proteins cannot be generalized; translational readthrough in itself does not require lengthy transcripts. If it does, then this requirement stems from some mechanism(s) specific to Drosophila species or insects. As the C-terminal regions of yeast TRC proteins are rich in residues of secondary structure and Pfam entities, the TR extensions could impair their stability and hence mark them for proteolytic degradation or modify their complex-forming properties. Additionally, their TR-derived extensions have reduced interaction capacities, in agreement with their structural features, such as well-structured nature, enrichment in hydrophobic amino acids and depletion in disorder-promoting amino acids. Although TR-derived protein segments have been shown to facilitate the peroxisomal localization of certain key enzymes in fungi^10^ and we also detected putative targeting motifs in some of the extensions, it seems improbable that targeting is the main role of TR in yeast. This is also supported by the data of Artieri and colleagues, who identified many orthologous TR genes with very different, often frameshifted extensions in their two yeast species^33^. Functional motifs are unlikely to be preserved in frameshifted regions, but, if the role of the extension was, for instance, to tune the lifetime of the protein, it could be achieved by the addition of very different sequences. Thus, the observed high sequence diversity of the TR extensions of orthologous yeast genes may suggests that if they fulfil any roles, those roles are independent of sequence motifs. It is also important to note, however, that the yeast species investigated by Jungreis *et al*. or Artieri *et al*. diverged much earlier than the 12 Drosophila species and thus the lack of conservation of yeast TR extensions does not necessarily imply that they are not functional. An in-depth analysis of the evolutionary conservation of these regions among *Saccharomyces cerevisiae* strains as well as closely related yeast species would be required to resolve this issue.

Based on our data and these considerations, we suspect that many yeast TR cases are non-functional cases of readthrough, which is also supported by the fact that their readthrough rates are considerably lower than those in *D. melanogaster*^30^. However, we also suggest a few potential roles for the functional TR cases: 1) since ribosomal components and translation initiation factors are largely overrepresented among the proteins affected by TR, it seems likely that TR somehow fine-tunes the function of ribosomes by altering the stability/lifetime or association properties of the subunits. This could introduce specialized ribosomes^55^ preferentially translating a subset of mRNAs or could affect translation fidelity or initiation/termination efficiency. If termination efficiency was affected by the TR extensions of ribosomal components, it would suggest that TR might be controlled by a positive or negative feedback loop. 2) Biosynthetic enzymes are also often affected by TR. Besides targeting them into certain cellular compartments^10^, TR extensions might simply impair the activity of these enzymes, as is the case for the high-affinity cAMP phosphodiesterase, PDE2^14^. Alternatively, TR extensions might fine-tune their activity, e.g. by adopting condition-dependent conformational states that affect the stability/activity of the enzyme in different ways.

The species-specific tendencies observed for the full candidate sets were also supported by the validated TR examples listed in Table 1. Finally, besides addressing the structure-function properties of TR proteins in the two species, we additionally identified orthologous pairs among them and evaluated the possibility of evolutionary conservation of their extensions. Neither sequence similarity nor the identified potential interaction motifs of the extensions support evolutionary conservation of TR between the identified orthologs. Most *D. melanogaster* TR candidates with yeast orthologs are not conserved even among Drosophila species^29^. In all, we do not see any evidence for the conservation of TR extensions between the two species, and we therefore propose that those appeared independently. In our opinion, the relatively large number of identified orthologs (almost 10% of the yeast candidates) can be explained if only a subset of proteins have properties compatible with TR. The fact that the median TR extension length was around 20 residues in both investigated species also implies that many extensions were either not affected by selection pressure at all, or did not have enough time to lose in-frame stop codons since they evolved into coding regions and became positively selected. Their complete lack of detectable protein domains and other Pfam entities can probably be attributed to the same reason.

In all, based on the results of our large-scale TR protein analysis, we conclude that TR most probably serves different purposes, if any, in yeast and fruit fly. While in *D. melanogaster* many proteins are affected by TR extensions and those seem to serve a very similar role to alternatively spliced exons, in yeast, where alternative splicing hardly exists, TR also seems to be more restricted, less conserved, affecting a markedly different subset of proteins and lacking clear functional hallmarks. In our view, these parallels with alternative splicing point to the basic differences between the complexity of the two species and thus further support the functional relevance of TR. The above mentioned considerations as well as the lack of detectable conservation in the extensions of orthologous TR proteins imply that the functions mediated by TR extensions are new in evolutionary terms, mainly specific to lower taxonomic levels (species, genus or families) and they could play an important role in species differentiation. We also denote however, that these suggestions need further investigation.

## Additional Information

**How to cite this article**: Pancsa, R. *et al*. Computational analysis of translational readthrough proteins in Drosophila and yeast reveals parallels to alternative splicing. *Sci. Rep.* **6**, 32142; doi: 10.1038/srep32142 (2016).

## Supplementary Material

Supplementary Information

Supplementary Dataset 1

Supplementary Dataset 2

Supplementary Dataset 3

Supplementary Dataset 4

Supplementary Dataset 5

Supplementary Dataset 6

Supplementary Dataset 7

## Figures and Tables

**Table 1:** Structural and interaction properties of validated eukaryotic TR-derived protein extensions.

Species	Gene name	Length CDS/TRE (res)	Disorder/low complexity/secondary structure/disordered binding site content of TRE (%)	Anchor sites in TRE	ELMs with predicted disorder per 100 residues unit length/(ELM patterns in TRE/overlap with disorder/Anchor sites)	ELMs in TRE with a low probability (<1E-03)	Ref.
S. cerevisiae	BSC4	131/107	4.7/0/44.3/0	0	0/(21/0/0)	TRG_NLS_MonoCore_2	13
S. cerevisiae	IMP3	183/80	12.6/0/54.4/0	0	6.3/(33/5/0)	DEG_APCC_DBOX_1, TRG_NLS_MonoExtC_3	9,13
S. cerevisiae	PDE2	526/22	0/0/90.5/0	0	0/(5/0/0)	LIG_SH3_4	14
U. maydis	PGK1	416/9	75.0/0/0/0	0	0/(1/0/0)	TRG_PTS1	10
U. maydis	ART1	418/7	50.0/0/0/0	0	0/(2/0/0)	LIG_PDZ_Class_3§	22
U. maydis	RPE1	311/9	100.0/0/0/0	0	44.4/(4/4/0)	TRG_PTS1	22
U. maydis	TPI1	248/9	50.0/0/0/0	0	0/(3/0/0)	TRG_PTS1	22
A. nidulans	GAPDH	336/14	84.6/0/0/0	0	21.4/(3/3/0)	LIG_PDZ_Class_1§	10
D. melanogaster	KEL	689/316	35.6/35.6/38.7 /33.7	8	14.6/(63/46/25)	12	15,18
D. melanogaster	HDC	649/430	64.8/43.1/39.2/43.4	11	28.4/(125/122/70)	5	16,17
D. melanogaster	OAF	332/154	66.7/64.7/28.8/36.0	3	41.6/(65/64/22)	6	56
D. melanogaster	SXL§	366/356	56.6/67.0/29.3/44.5	11	37.1/(132/132/76)	4	57
D. melanogaster	SYN	537/444	51.9/35.7/19.6/49.2	10	41.7/(187/185/104)	15	12
O. cuniculus	HBB1	147/23	27.3/0/4.6/0	0	0/(2/0/0)	—	8,11
H. sapiens	MPZ	248/63	85.5/17.7/30.1/69.4	2	25.4/(16/16/13)	LIG_MYND_1	19
H. sapiens	OPRK1	380/29	21.4/0/42.9/0	0	0/(11/0/0)	LIG_PDZ_Class_1	26,29
H. sapiens	OPRL1	370/29	82.1/0/17.9/0	0	62.1/(18/18/0)	DEG_SCF_FBW7_2, LIG_PDZ_Class_1	26,29
H. sapiens	MAPK10	464/14	15.4/0/30.8/100.0	1	0/(2/0/2)	LIG_PTB_Apo_2	26
H. sapiens	AQP4	323/29	53.6/0/14.3/35.7	1	17.2/(6/5/3)	LIG_PDZ_Class_3	21,26
H. sapiens	VEGFA	232/22	66.7/0/23.8/0	0	54.5/(12/12/0)	—	20
H. sapiens	SYTL2	934/52	0/0/50.1/0	0	9.6/(20/5/0)	LIG_PDZ_Class_2, MOD_LATS_1	21
H. sapiens	CACNA2D4	1137/5	100.0/0/0/0	0	0/(0/0/0)	—	21
H. sapiens	MDH1	334/19	16.7/0/38.9/0	0	0/(4/0/0)	TRG_PTS1	22,30
H. sapiens	LDHB	334/7	66.7/0/0/0	0	0/(6/0/0)	TRG_PTS1, LIG_PDZ_Class_1	21,22
H. sapiens	EDEM3	932/30	27.6/0/48.3/31.0	1	23.3/(9/7/8)	TRG_NLS_MonoExtC_3	21
H. sapiens	AGO1	857/34	78.8/0/30.3/18.2	1	23.5/(8/8/2)	LIG_14–3–3_1	20
H. sapiens	MTCH2	303/11	10.0/0/30.0/0	0	0/(1/0/0)	LIG_PDZ_Class_1	20

^§^The previously reported functional peroxisome targeting signals were not detected due to their unconventional sequence pattern.
