# Supplementary material for: Computational analysis of translational readthrough proteins in Drosophila and yeast reveals parallels to alternative splicing
Source: Sci Rep. 2016 Aug 26;6:32142. doi: 10.1038/srep32142 (PMC4999894; doi:10.1038/srep32142)
Supplement: Supplementary Information [file srep32142-s1.pdf]

Supplementary information

Computational analysis of translational readthrough proteins in Drosophila and yeast reveals parallels to alternative splicing

Rita Pancsa[1]*, Mauricio Macossay-Castillo[1], Simone Kosol[1], Peter Tompa[1,2]

1- Flanders Institute for Biotechnology (VIB), Structural Biology Research Center, Vrije Universiteit Brussel, 1050 Pleinlaan 2, Brussels, Belgium

2- Institute of Enzymology, Research Centre for Natural Sciences, Hungarian Academy of Sciences, 1117 Budapest, Magyar tudósok körútja 2.

***Correspondence to** Rita Pancsa (email: ritapancsa@gmail.com).

Present Address: Rita Pancsa, MRC Laboratory of Molecular Biology, Cambridge, CB2 0QH, United Kingdom

Supplementary Figures:

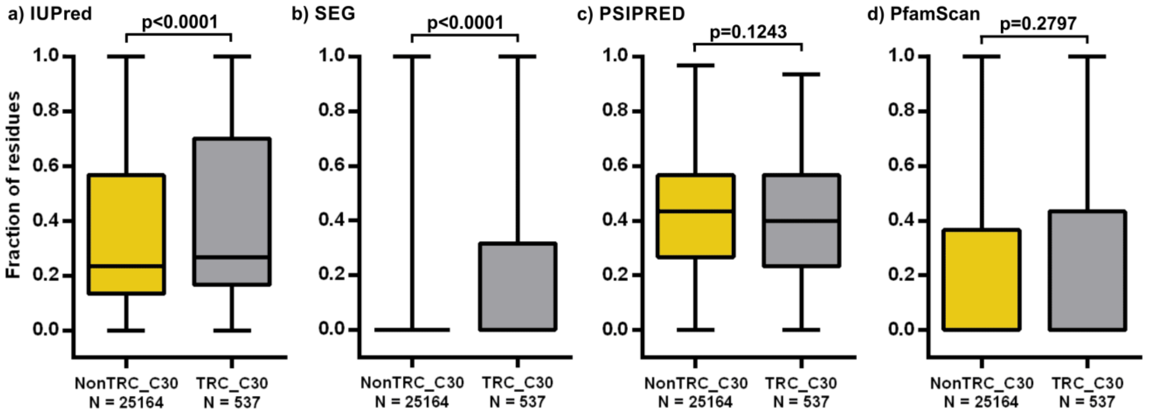

Figure S1. The structural properties of the C-termini of D. melanogaster TR candidates.

The fractions of residues in a) disordered (by IUPred), b) low sequence complexity (by SEG), c) secondary structure (by PSIPRED) and d) domain (by PfamScan) regions were calculated for the last 30-residue segments of fruit fly TR candidates (TRC_C30; grey) and compared to those of non-candidates (NonTRC_C30; yellow) using two-tailed Mann-Whitney U tests. P-values are indicated. The significance threshold was set to p=0.0125 by Bonferroni correction.

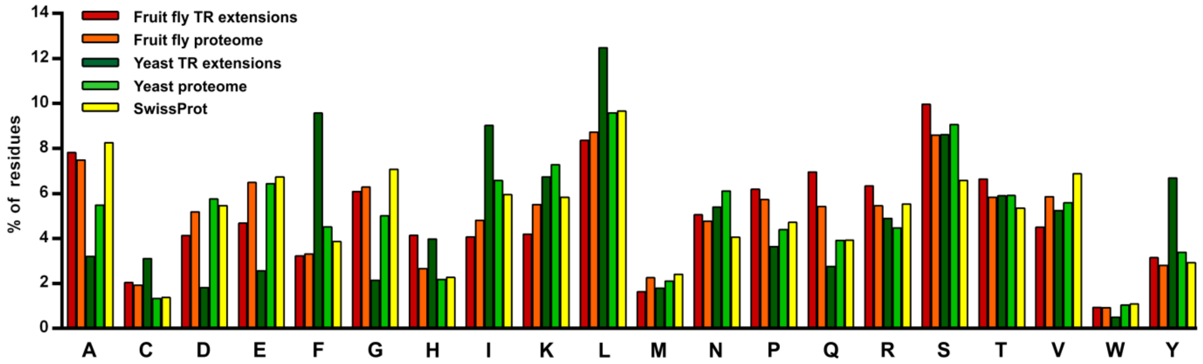

Figure S2. The amino acid compositions of TR extensions in the investigated species.

The amino acid compositions of fruit fly and yeast TR extensions are compared to that of the corresponding reference proteomes and SwissProt (04/2014).

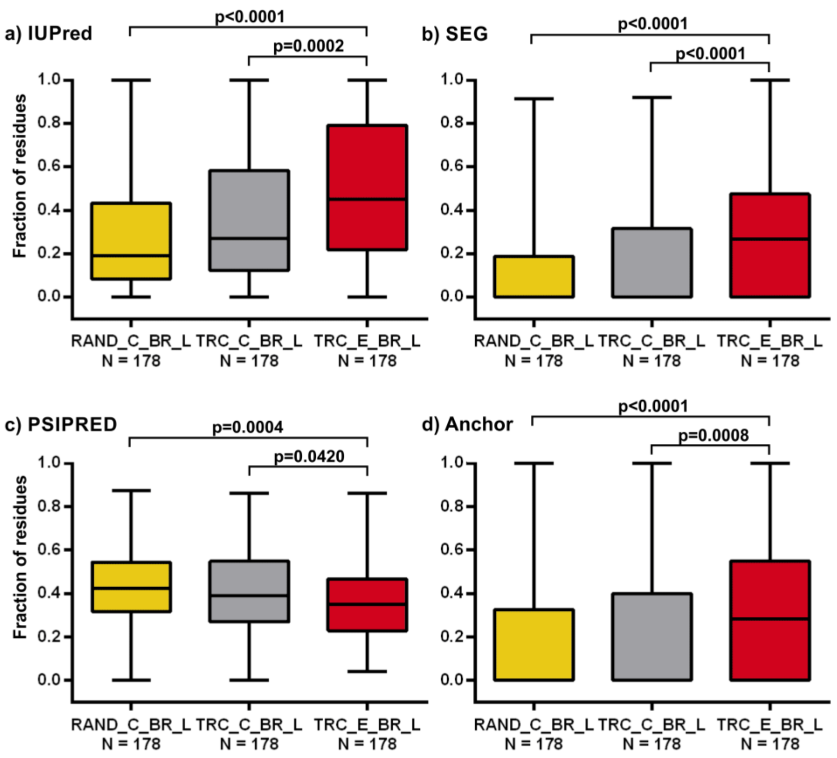

Figure S3. The structural and interaction properties of biologically relevant D. melanogaster TR extensions.

Long TR extensions were filtered for displaying evolutionary conservation or biologically relevant readthrough rates (>1.2% of the CDS). The fractions of residues in a) disordered, b) low complexity, c) secondary structure and d) disordered binding regions were compared between the extensions (TRC_E_BR_L, in red) and the two similarly filtered reference segment sets, RAND_C_BR_L (in yellow) and TRC_C_BR_L (in grey) by Kruskal-Wallis test coupled with Dunn's multiple comparison test. The p-values indicated above the boxes are adjusted to the number of comparisons performed in each panel. The significance threshold is adjusted to p=0.0125 by Bonferroni correction.

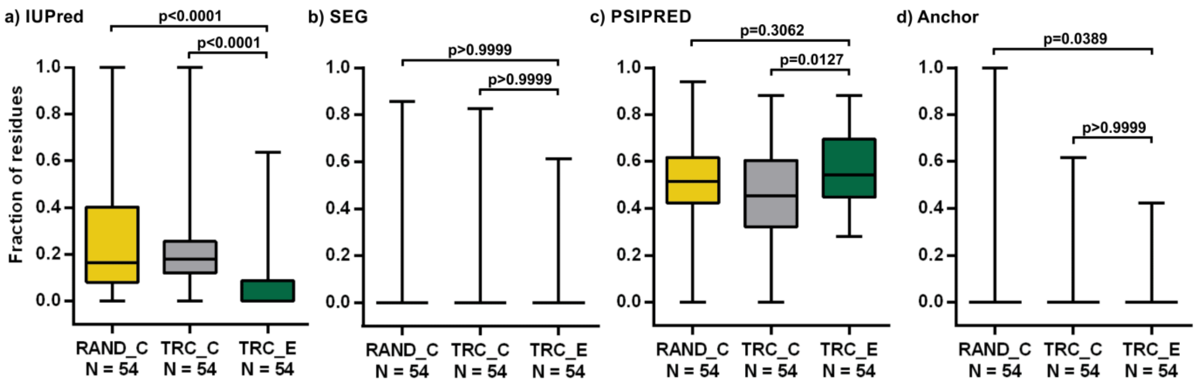

Figure S4. The structural and interaction properties of long yeast TR extensions.

The predicted structural and interaction properties of long TR extensions (>25 residues; TRC_E_L, in red) were compared to those of the similarly filtered two reference segment sets, RAND_C_L (in yellow) and TRC_C_L (in grey). The fractions of residues in a) disordered, b) low complexity, c) secondary structure, d) and disordered binding regions were used for comparisons applying Kruskal-Wallis test coupled with Dunn's multiple comparison

post-hoc test. The p-values are adjusted to the number of comparisons performed in each panel. The significance threshold is decreased to p=0.0125 by Bonferroni correction due to the multiplicity of compared properties.

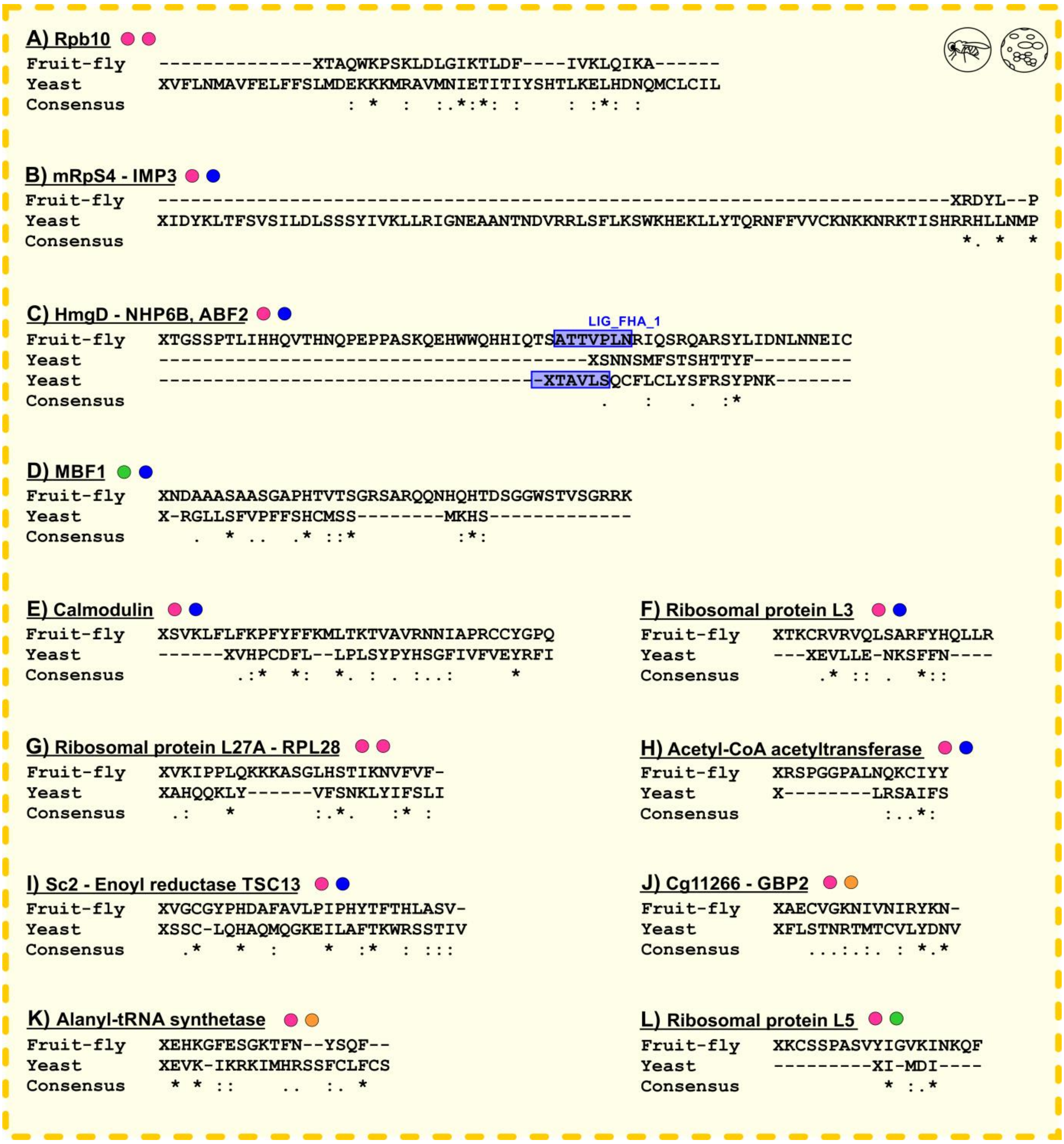

Figure S5. Sequence and motif conservation within the extensions of orthologous TR protein pairs.
Orthologous TR protein pairs were identified among the candidates and aligned using ClustalW. If the starts of the extensions were not well-fitted in the resulting alignments, the extensions were separately aligned. For each pair, the fitted extension regions are shown together with the corresponding ClustalW consensus patterns (since the X represents ambiguous amino acids, the corresponding consensus stars were removed). In the 12 pairs of extensions we only detected one potentially conserved extension ELM (after excluding motif types of high probability scores (>1E-03)), which is shown in blue. The colour of the first circle next to the protein names represents the conservation of TR in the given gene in the 12 Drosophila species (pink – no, green – yes). The colour of the second

circle indicates the level of conservation of TR between the two yeast species investigated by Artieri et al.; pink – no, blue – yes but frameshifted, orange – yes but poorly conserved, green – yes and well conserved).

Supplementary Tables:

Supplementary Tables S1-6 and 9 are available as separate excel files.

Table S7. Comparisons between the structural properties of TR extensions and equivalent reference C-termini on the residue level.

Datasets	Structurally disordered residues (IUPred short)	Residues in low sequence complexity (SEG)	Residues in secondary structure (PSIPRED)	Residues in domains (PfamScan)	Residues in disordered binding sites (Anchor)
**Fly TRC_E^{OBS} vs. fly RAND_C^{EXP} (N = 18971)**	9378 vs. 6714; chi^2 = 1635.4; p<1E-08	4719 vs. 2136; chi^2 = 3518.5; p<1E-08	6565 vs. 7733; chi^2 = 297.6; p<1E-08	0 vs. 4379; p<1E-08	6274 vs. 3791; chi^2 = 2031.6; p<1E-08
**Fly TRC_E^{OBS} vs. fly TRC_C^{EXP} (N = 18971)**	9378 vs. 7474; chi^2 = 799.9; p<1E-08	4719 vs. 2897; chi^2 = 1351.7; p<1E-08	6565 vs. 7659; chi^2 = 261.8; p<1E-08	0 vs. 5021; p<1E-08	6274 vs. 3972; chi^2 = 1686.7; p<1E-08
**Yeast TRC_E^{OBS} vs. yeast RAND_C^{EXP} (N = 3413)**	455 vs. 1078; chi^2 = 525.4; p<1E-08	281 vs. 261; chi^2 = 1.578; p=0.2090	1728 vs. 1636; chi^2 = 9.829; p=0.001718	0 vs. 622; p<1E-08	182 vs. 374; chi^2 = 110.1; p<1E-08
**Yeast TRC_E^{OBS} vs. yeast TRC_C^{EXP} (N = 3413)**	455 vs. 1128; chi^2 = 598.9; p<1E-08	281 vs. 373; chi^2 = 25.2; p=5.2E-07	1728 vs. 1514; chi^2 = 54.11; p<1E-08	0 vs. 1397; p<1E-08	182 vs. 258; chi^2 = 23.9; p=1.01E-06

Table S8. Comparisons between the interaction properties of TR extensions and equivalent reference C-termini.

Datasets	Residues in exposed ELMs	Residues in LIG ELMs	Residues in MOD ELMs	Residues in CLV ELMs	Residues in TRG ELMs	Residues in DEG ELMs	Residues in DOC ELMs
**Fly TRC_E^{OBS} vs. fly RAND_C^{EXP} (N = 18971)**	9036 vs. 7006; chi^2 = 932.149; p<1E-08	3797 vs. 3039; chi^2 = 224.83; p<1E-08	6438 vs. 4544; chi^2 = 1037.54; p<1E-08	584 vs. 496; chi^2 = 15.85; p=6.857E-05	753 vs. 815; chi^2 = 4.849; p=0.0277	164 vs. 188; chi^2 = 2.967; p=0.08498	2139 vs. 1510; chi^2 = 284.22; p<1E-08
**Yeast TRC_E^{OBS} vs. yeast RAND_C^{EXP} (N = 3413)**	360 vs. 727; chi^2 = 234.771; p<1E-08	125 vs. 261; chi^2 = 76.171; p<1E-08	225 vs. 454; chi^2 = 132.65; p<1E-08	20 vs. 47; chi^2 = 15.15; p=9.93E-05	63 vs. 121; chi^2 = 28.329; p=1E-07	9 vs. 7; chi^2 = 0.322; p=0.5704	106 vs. 131; chi^2 = 4.765; p=0.02904
